# Technical Note: Investigation of the dosimetric impact of stray radiation on the Common Control Unit of the IBA Blue Phantom^2^

**DOI:** 10.1002/acm2.12769

**Published:** 2019-11-15

**Authors:** Guoqiang Cui, Jun Duan, Yun Yang, Fang‐Fang Yin

**Affiliations:** ^1^ Department of Radiation Oncology Duke University Medical Center Durham NC USA

**Keywords:** Common Control Unit (CCU), IBA Blue Phantom^2^, inverse square law, stray radiation

## Abstract

**Purpose:**

This technical note aims to investigate the dosimetric impact of stray radiation on the Common Control Unit (CCU) of the IBA Blue Phantom^2^ and the measured beam data.

**Methods:**

Three CCUs of the same model were used for the study. The primary test CCU was placed at five distances from the radiation beam central axis. At each distance, a set of depth dose and beam profiles for two open and two wedge fields were measured. The field sizes were 10 × 10 cm^2^ and 30 × 30 cm^2^ for the open fields, and 30 × 30 cm^2^ and 15 × 15 cm^2^ for the 30° and 60° wedges, respectively. The other two CCUs were used to cross check the data of the primary CCU. Assuming the effect of stray radiation on the data measured at the farthest reachable distance 4.5 m is negligible, the dosimetric impact of stray radiation on the CCU and consequently on the measured data can be extracted for analysis by comparing it with those measured at shorter distances.

**Results:**

The results of three CCUs were consistent. The dosimetric impact of stray radiation was greater for lower energies at larger field sizes. For open fields, the data variation was up to 4.5% for depth dose curves and 7.1% for beam profiles. For wedge fields, the data variation was up to 9.3% for depth dose curves and 10.6% for beam profiles. Moreover, for wedge field profiles in the wedge direction, they became flatter as the CCU was placed closer to the primary radiation beam, manifesting smaller wedge angles.

**Conclusion:**

The stray radiation added a uniform background noise on all measured data. The magnitude of the noise is inversely proportional to the square of the distance of the CCU to the primary radiation beam, approximately following the inverse square law.

## 1 INTRODUCTION

Three‐dimensional (3D) water scanners are routinely used for commissioning and quality assurance (QA) of radiotherapy linear accelerators and treatment planning systems.^1^, ^2^, ^3^ The Blue Phantom^2^ (IBA Dosimetry GmbH, Schwarzenbruck, Germany) is widely used for measurement and analysis of the radiation beams of medical linear accelerators.^4^, ^5^ It consists of a Common Control Unit (CCU) and a water phantom with three‐dimensional servo. The CCU integrates a controller and two independent electrometers. It also has built‐in pressure and temperature sensor interfaces which automatically apply the temperature and pressure correction factor.^6^

In the User’s Guide, the manufacturer states that “the CCU is a sensitive electronic device that can be affected by stray radiation. In order to prevent significant influence of scattered radiation on the electronics and to increase the lifetime of the CCU, it has to be placed at a minimum distance of 3 m from the radiation field border.”^6^ Many users consider this recommendation as a protection against radiation damage to the CCU. Few realize that the distance of the CCU to the primary radiation beam can significantly affect the measurement results if the recommendation of minimum distance is not followed. We discovered inadvertently that if the CCU is positioned close to the primary radiation beam, it can result in as high as 10.6% of discrepancies in the measurement results. If users are unaware of this adverse effect and use the tainted measurement results for commissioning treatment planning systems, it can result in substantial systematic errors. It also can cause inconsistency and confusion in linear accelerator commissioning and annual quality assurance.^4^, ^5^, ^7^ The purpose of this study is to provide a systematic assessment of the adverse effect of the CCU when it is placed at various distances from the primary radiation, and to make users aware of the dosimetric impact of stray radiation on the measurement results if the manufacturer’s recommendation is not followed.

## 2 MATERIALS AND METHODS

### 2.1 Data measurement

**Figure 1:**
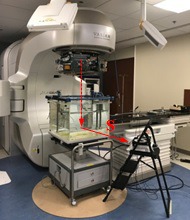
Setup of the Common Control Unit (CCU) during the data measurement. The CCU was placed on a stand at 82 cm above the floor. The stand was then positioned at various distances S from the radiation beam. S is the distance between the rear edge of the CCU and the radiation beam central axis. The geometries used to measure the data were gantry = 0°, collimator = 0°, and SSD = 100 cm.

Three CCUs of the same model were used in the study. One unit CCU1, served as the primary unit, was used to collect all the data; and the other two, CCU2 and CCU3, were used to cross check the results of the primary unit to rule out the possibility that the adverse effect was isolated to a specific CCU. During the data collection, the CCUs were placed on a stand (82 cm above the floor) rather than on the couch to minimize the ambient scattering. It also allowed us to position the CCU to a farther distance up to 4.5 m. The stand was then positioned at various distances from the radiation field central axis, as shown in Fig. 1. The primary CCU was placed at distances of 0.5, 1.0, 2.0, 3.0, and 4.5 m from the radiation beam central axis to the rear edge of the unit. At each distance S, a set of depth dose and beam profiles at the depth of 10 cm for two open and two wedge fields were collected for analysis. The field sizes were 10 × 10 cm^2^ and 30 × 30 cm^2^ for the open fields, and 30 × 30 cm^2^ and 15 × 15 cm^2^ for the 30° and 60° wedges, respectively.

All data were measured in water with the IBA Blue Phantom^2^ using a Varian TrueBeam STx linac. Both the field and the reference detectors were 0.125 cc cylindrical ion chambers (Model CC13, IBA Dosimetry GmbH, Schwarzenbruck, Germany). Two photon energies 6 and 15 MV were used for the data measurement. During the measurement, the reference detector was placed above the jaws so that its signal was not affected by field sizes. The field detector relative to the reference detector was normalized to 100% only once using the 6 MV photon beam with the field detector placed in the central beam at the depth of 1.5 cm of a 10 × 10 cm^2^ open field, while the CCU was placed at the distance S = 0.5 m. This normalization was then kept unchanged throughout the data measurement at all distances, field sizes, and energies.

### 2.2 Data processing and analysis

The data were processed and analyzed with OmniPro Accept 7.5 (IBA Dosimetry GmbH, Schwarzenbruck, Germany). The dosimetric impact of stray radiation on the CCU at various distances was reflected on the measured depth dose curves and beam profiles. The data measured at different distances were compared and examined. For open fields of 30 × 30 cm^2^ and 10 × 10 cm^2^ field sizes, the depth dose curves and beam profiles at distances of 0.5, 1.0, 2.0, 3.0, and 4.5 m were plotted on a single graph for comparison. For a given photon energy, the depth dose and beam profiles of two field sizes were compared. For a given field size, the depth dose and beam profiles for two photon energies 6 and 15 MV were also compared. For wedge fields, 30 × 30 cm^2^ field for the 30° wedge and 15 × 15 cm^2^ field for the 60° wedge, the depth dose and beam profiles at distances of 0.5, 1.0, 2.0, and 3.0 m were compared.

## 3 RESULTS

### 3.1 Dosimetric impact of stray radiation on the depth dose and beam profiles of open fields

**Figure 2:**
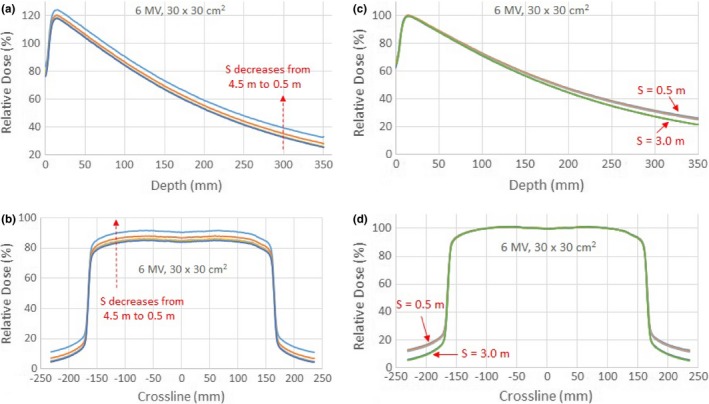
Depth dose curves (a), and crossline beam profiles (b), of the 30 × 30 cm^2^ field for 6 MV photon beam with the test Common Control Unit (CCU) placed at S = 0.5, 1.0, 2.0, 3.0, and 4.5 m from the radiation beam central axis. Depth dose curves (c) and crossline beam profiles (d) of the 30 × 30 cm^2^ field for 6 MV photon beam for all three CCUs placed at the distances S = 0.5 and 3.0 m from the radiation beam central axis.

Figures 2(a) and 2(b) show the depth dose curves and crossline beam profiles of a 30 × 30 cm^2^ field for 6 MV photon beam with the primary CCU placed at five different distances. The inline beam profiles showed the same effect as the crossline beam profiles. To keep it concise, we present only the results of the crossline scans. The raw measured curves in the figures were not rescaled to demonstrate that the background noise increased as the CCU was positioned closer to the primary radiation beam. The only variable in these depth dose curves and beam profiles was the distance of the CCU to the primary radiation beam. The variations due to the distance factor were up to 7.1% in the depth dose curves and 6.8% in the beam profiles. This demonstrated that the performance of the CCU was affected by the stray radiation and the effect was distance dependent.

The same measurements were repeated with CCU2 and CCU3. The percentage depth dose curves and beam profiles at two distances S = 0.5 and 3.0 m for all three CCUs are shown in Figs. 2(c) and 2(d). The fact that the depth dose curves and crossline beam profiles for the three CCUs completely overlap demonstrates that the stray radiation effect was consistent for all CCUs, which rules out the possibility that it was the behavior of a specific CCU.

**Figure 3:**
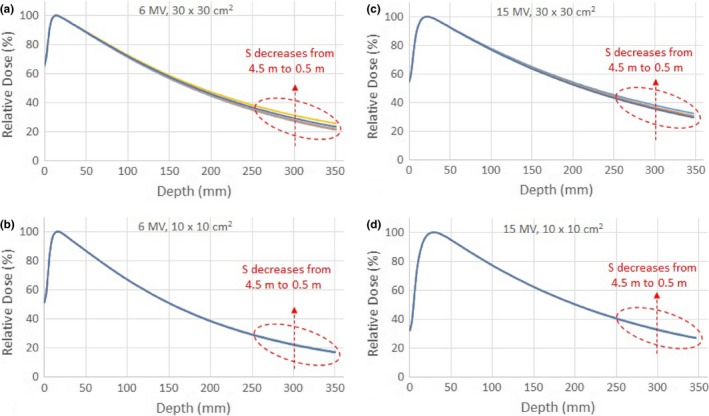
PDDs of the 30 × 30 cm^2^ field (a) and 10 x 10 cm^2^ field (b) for 6 MV photon beam; PDDs of the 30 x 30 cm^2^ field (c) and 10 x 10 cm^2^ field (d) for 15 MV photon beam, respectively, with the test Common Control Unit (CCU) placed at distances S = 0.5, 1.0, 2.0 , 3.0, and 4.5 m from the radiation beam central axis.

To examine the data in a clinical perspective, the raw depth dose curves were normalized to 100% at the dose maximum to yield PDDs. Two photon energies at two different field sizes were compared. For 6 MV photon beams, Figs. 3(a) and 3(b) show the PDDs of the 30 × 30 cm^2^ and 10 × 10 cm^2^ field sizes, respectively. As one can see that in the figures, the effect of the stray radiation on the CCU was stronger with larger field size, resulting in greater discrepancies in PDDs. The dose variations in the PDDs of the 30 × 30 cm^2^ field size for 6 MV photon beam were up to 4.5% at the deepest depth. Figs. 3(c) and 3(d) show the same set of data for 15 MV photon beam and the same conclusion can be drawn. Furthermore, by comparing Figs. 3(a) with 3(c), one can see that, for the same field size 30 × 30 cm^2^, the effect of stray radiation was stronger with lower energy, as lower energy X‐rays generate more scatter radiation. But for the field size 10 × 10 cm^2^, this effect was less prominent for both energies, as shown in Figs. 3(b) and 3(d).

**Figure 4:**
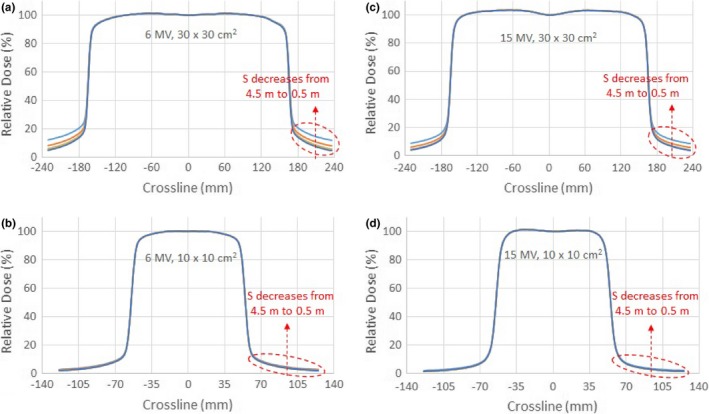
Crossline beam profiles of the 30 x 30 cm^2^ field (a) and 10 x 10 cm^2^ field for 6 MV photon beam (b); Crossline beam profiles of the 30 x 30 cm^2^ field (c) and 10 x 10 cm^2^ field for 15 MV photon beam (d), respectively, with the test Common Control Unit placed at distances S = 0.5, 1.0, 2.0, 3.0, and 4.5 m from the radiation beam central axis.

Figures 4(a) and 4(b) show the crossline beam profiles of the 30 × 30 cm^2^ and 10 × 10 cm^2^ 6 MV photon beams normalized to 100% at the central axis, respectively. Figures 4(c) and 4(d) show the same set of data for 15 MV photon beams. The stray radiation effect of the CCU was manifested at the tails of the normalized beam profiles. The dose discrepancies at the tails due to the CCU effect were up to 7.1% of the central axis dose, which was 131% relative to dose at the point without the CCU effect. The dependence on the field size and energy is similar to that of the PDDs, that is, the effect of stray radiation on the CCU was greater for lower energies at larger field sizes. Moreover, the dose variations were negligibly small (<0.4%) for distances beyond 3.0 m.

### 3.2 Dosimetric impact of stray radiation on the depth dose and beam profiles of wedge fields

**Figure 5:**
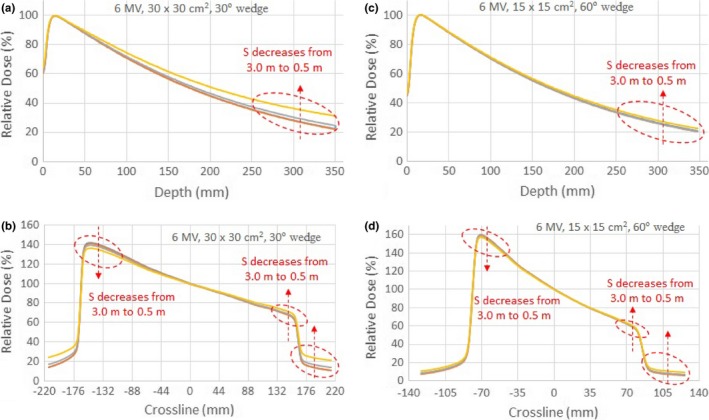
PDDs (a) and crossline beam profiles (b) of the 30 x 30 cm^2^ field for the 30° wedge; PDDs (c) and crossline beam profiles (d) of the 15 x 15 cm^2^ field for the 60° wedge; respectively, for 6 MV photon beam with the test Common Control Unit placed at distances S = 0.5, 1.0, 2.0, and 3.0 m from the radiation beam central axis.

For wedge fields, we present only the data measured for 6 MV photon beam because the data measured for 15 MV showed similar trend but the distance effect was less prominent. Figures 5(a) and 5(b) show the PDDs and crossline beam profiles of the 30 × 30 cm^2^ field for the 30° wedge. The PDDs and the beam profiles were normalized to 100% at the dose maximum and the beam central axis, respectively. As compared with the PDDs of the same field size for the open field, the dose variation due to CCU effect for the wedge field was greater, up to 9.3% of the dose maximum, as compared with 4.5% for the open field as shown in Fig. 2(a). This is likely due to increased scattering from the wedge. The CCU effect on the profiles in the wedge direction manifested as decrease in wedge angles as the CCU was positioned closer to the primary radiation beam as shown in Fig. 5(b). The variations due to CCU effect were about −5.0% at the toe, 4.8% at the heel, and 10.6% at the tail. Figures 5(c) and 5(d) show the PDDs and profiles of the 15 × 15 cm^2^ field for the 60° wedge. They showed the same trend as the 30° wedge. But due to the smaller field size, the impact on the PDDs and profiles was less prominent than that for the 30 × 30 cm^2^ field of the 30° wedge.

## 4 DISCUSSION

**Figure 6:**
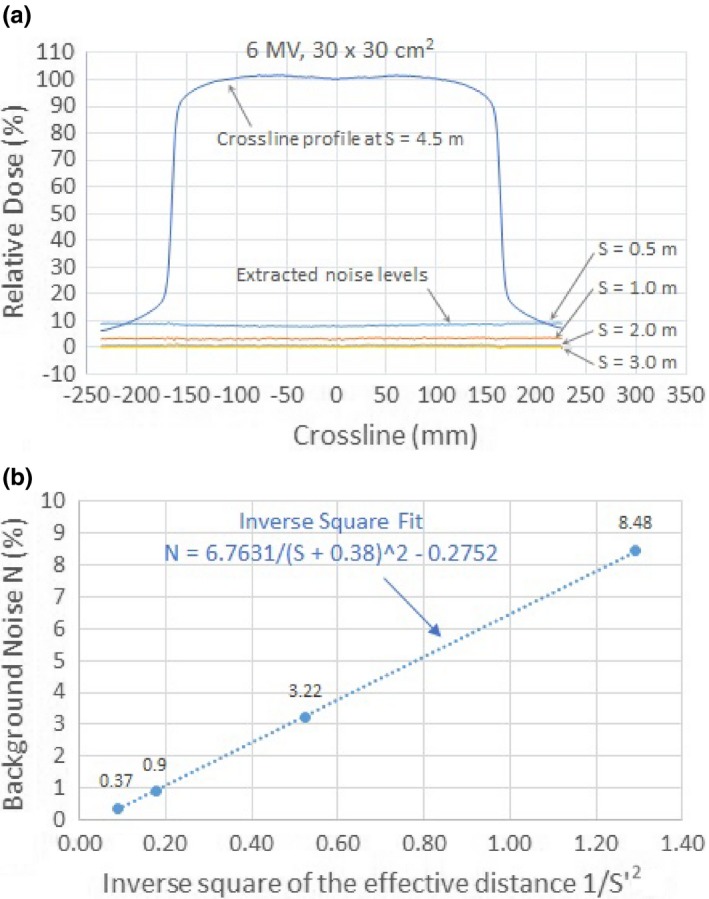
(a) Top curve is the crossline profile of the 30 x 30 cm field for 6 MV photon beam at S = 4.5 m. Lower lines are the extracted background noise levels. (b) The inverse‐square fit of the background noise levels. The solid dots are the averaged extracted noise levels and the dashed line is the inverse square fit.

We have investigated the effect of stray radiation on the CCU of the IBA Blue Phantom^2^ and its dosimetric impact on the measured depth dose and beam profiles. Three CCUs of the same model were used in the study and yielded consistent results. As shown in Figs. 2(a) and 2(b), the stray radiation effectively added a background “noise” to the measured data that increased as the CCU was positioned closer to the primary radiation beam. Assuming the effect of stray radiation on the data measured at the farthest distance of 4.5 m was negligible, we extracted the background noise levels, for example, by subtracting the profiles of the 30 × 30 cm^2^ field for 6 MV open beam measured at S = 4.5 m from those measured at 0.5, 1.0, 2.0, and 3.0 m, respectively. Figure 6(a) shows an example of the beam profile at S = 4.5 m and the extracted background noise levels. The relatively flat background noise levels indicated that the noise was uniform across the field range throughout the measurement.

To quantify the noise level, we defined an averaged noise level N by averaging the magnitude of the extracted noise level across the measured field range. The averaged noise level N as a function of the distance S of the CCU to the central axis of the primary radiation was fitted to N = a/(S + c)^2^ + b, where a = 6.7631, b = −0.2752, and c = 0.38 m are fitting parameters. All the fitting parameters are listed in Table 1 and the fitting curve is plotted in Fig. 6(b). The vertical axis is the averaged noise level N and the horizontal axis is the inverse square of an effective distance S' = S + c. The effective distance was used due to the fact that both the virtual source of the stray radiation and the exact location in the CCU affected by the stray radiation were unknown. The solid dots are the averaged extracted noise levels and the dashed line is the fitted curve. It should be noted that the difference between the profiles measured at 3.0 and 4.5 m was small (0.37%), suggesting that the vendor's recommendation of placing the CCU at least 3.0 m from the primary radiation is sufficient to mitigate such an effect. The mechanism of the stray radiation effect on the CCU is unknown. Further investigation is warranted to find the cause of this effect. To further reduce the stray radiation effect, we recommend that the manufacturer better shield the CCU from stray radiation, or allow to place the CCU outside the treatment vault.

**Table 1:** Fitting parameters for the inverse square fit of the noise level N = a/(S + c)^2^ + b, where a = 6.7631, b = −0.2752, and c = 0.38 m. S is the distance of the Common Control Unit to the central axis of the primary radiation. S' = S + 0.38 is the effective distance.

S (m)	S' = S + 0.38 (m)	Inverse square of the effective distance 1/S'^2^	Averaged noise level N	Fitted noise level N
3.0	3.38	0.09	0.37	0.32
2.0	2.38	0.18	0.90	0.92
1.0	1.38	0.53	3.22	3.28
0.5	0.88	1.29	8.48	8.46

## 5 CONCLUSION

Stray radiation can have a significant impact on the performance of the CCU, manifested as a uniform background noise added to the measured data. The averaged magnitude of the added background noise level is inversely proportional to the square of the distance of the CCU to the primary radiation beam, approximately following the inverse square law. The adverse dosimetric impact on the measured depth dose and beam profiles can be substantial if the recommended minimum distance is not met. It is important that users are aware of the impact of the CCU effect and always follow the manufacturer’s recommendation to place the CCU at a minimum distance of 3 meters from the primary radiation beam.

## CONFLICT OF INTEREST

The authors declare no conflict of interest.
